# Fluid Extracts Made with Acetic Acid

**Published:** 1899-10

**Authors:** 


### THERAPEUTICAL AND PHARMACEUTICAL NOTES.

UNDER THE DIRECTION OF W. J. MARTIN, M.D.C.,
KANKAKEE, ILL.

FLUID EXTRACTS MADE WITH ACETIC ACID.—During the past year we have had samples of fluid extract of various drugs submitted to us for inspection and also for internal administration in veterinary practice. These fluid extracts differed quite radically from the ordinary fluid extracts of the *United States Pharmacopœia*, insomuch as the extracting medium employed consisted not of the usual dilute alcoholic menstruum, but a 10 per cent. solution of acetic acid in water.

In appearances these acetic fluid extracts, or fluid "acetracts," as they have been named by some, are of a rich, dark color, having a distinct acetous taste and odor. The keeping qualities of these new extracts appear to be very good. Samples of them after standing for nearly a year show but little precipitation of the solid matter held in suspension.

The fluid extract of aconite prepared by this method after standing for nearly a year remained perfectly free from sedimentation. The fluid extract of belladonna showed a small amount of sedimentation, but scarcely as much as might be seen in any fluid extract prepared with an alcoholic menstruum. The fluid extract of digitalis, made from the leaves, showed the largest amount of sediment of any of the acetic fluid extracts experimented with.

As therapeutic agents we have given these acetic fluid extracts a very careful trial, and in each and every instance have found them to be as physiologically active and fully as reliable as the best fluid extracts prepared with alcohol. This is nothing more than what should be expected, because it is well known that acetic acid has as great, if not greater, extracting properties than grain alcohol.

The question has of late been frequently asked, Do the acetic fluid extracts contain enough acetic acid to render them therapeutically objectionable? To this we can answer that, so far as our experience has gone, no deleterious effects whatever need be apprehended from the small amount of acetic acid contained in each dose of these fluid extracts.

In diluting with water for dispensing purposes the acetic fluid extracts have a decided advantage over the alcoholic extracts in that the former do not present such a cloudy or "muddy" appearance as the latter do. In this dilute condition the acetic taste and odor are scarcely perceptible.

It is not to be supposed that acetic acid can entirely supplant alcohol in the preparation of all medicinal fluid extracts, but it has been asserted by an eminent professor of pharmacy that one-half of the official fluid extracts of the *United States Pharmacopœia* can be successfully prepared by a menstruum containing 10 per cent. of acetic acid in water.

Of course, the only object in substituting acetic acid for alcohol as an extracting medium in preparing fluid extract is on the score of economy. There is no reason why, in the nature of things, if therapeutically fluid extracts made with acetic acid are the equal of alcoholic extracts, a vast saving will not be effected. As showing the difference in price between the two fluid extracts, I quote below from a recent price-list:

| *Fluid Extracts made with Alcohol.* | | *Fluid Extracts made with Acetic Acid.* | |
|---|---|---|---|
| Fluid extract of— | Pound. | Fluid extract of— | Pound. |
| Belladonna, leaf . . | $ .81 | Belladonna, leaf . . | $ .31 |
| Belladonna, root . . | 1.04 | Belladonna, root . . | .53 |
| Gentian . . . | .63 | Digitalis . . . | .35 |
| Digitalis . . . | .81 | Gentian . . . | .40 |
| Nux Vomica . . | .89 | Nux vomica . . . | .31 |
| Cannabis Indica . . | 1.04 | Cannabis Indica . . | .58 |

From a perusal and a comparison of the above table it will be seen that a considerable saving can be effected in practice by using fluid extracts made with acetic acid. The most objectionable feature which the author finds in using acetic fluid extracts is that in cold weather they freeze at about the same temperature as water. Whether this freezing impairs the therapeutic efficiency of the extract yet remains to be seen. The author submitted samples of the acetic fluid extract to a freezing temperature until frozen solid, and when heat was applied and the extracts again became liquid they appeared as clear as before freezing.

On the whole, we see no reason why fluid extracts made with acetic acid should not form an important part of the armamentarium of the veterinarian.

---

Dr. Robert Ward, of Baltimore, is the veterinary representative on the State Board of Horseshoers of Maryland; Dr. Thomas M. Quinn, of Astoria, L. I., of the Empire State; Dr. M. Wilson, of Mendota, of the Illinois board.

